# Genetic variation in vitamin D-related genes and risk of colorectal cancer in African Americans

**DOI:** 10.1007/s10552-014-0361-y

**Published:** 2014-02-23

**Authors:** Fabio Pibiri, Rick A. Kittles, Robert S. Sandler, Temitope O. Keku, Sonia S. Kupfer, Rosa M. Xicola, Xavier Llor, Nathan A. Ellis

**Affiliations:** 1 Department of Pediatrics and the Institute of Human Genetics, University of Illinois at Chicago, 900 S. Ashland Ave. MC 767, Chicago, IL 60607 USA; 2 Department of Medicine and the Institute of Human Genetics, University of Illinois at Chicago, Chicago, IL USA; 3 Division of Gastroenterology and Hepatology, Department of Medicine, University of North Carolina, Chapel Hill, NC USA; 4 Department of Medicine, Section of Gastroenterology, Hepatology and Nutrition, University of Chicago Medicine, Chicago, IL USA

**Keywords:** Vitamin D, Colorectal cancer, Genetic risk factors, Cancer health disparities, African American population

## Abstract

**Purpose:**

Disparities in both colorectal cancer (CRC) incidence and survival impact African Americans (AAs) more than other US ethnic groups. Because vitamin D is thought to protect against CRC and AAs have lower serum vitamin D levels, genetic variants that modulate the levels of active hormone in the tissues could explain some of the cancer health disparity. Consequently, we hypothesized that genetic variants in vitamin D-related genes are associated with CRC risk.

**Methods:**

To test this hypothesis, we studied 39 potentially functional single-nucleotide polymorphisms (SNPs) in eight genes (*CYP2R1*, *CYP3A4*, *CYP24A1, CYP27A1*, *CYP27B1, GC, DHCR7*, and *VDR*) in 961 AA CRC cases and 838 healthy AA controls from Chicago and North Carolina. We tested whether SNPs are associated with CRC incidence using logistic regression models to calculate *p* values, odds ratios, and 95 % confidence intervals. In the logistic regression, we used a log-additive genetic model and used age, gender, and percent West African ancestry, which we estimated with the program STRUCTURE, as covariates in the models.

**Results:**

A nominally significant association was detected between CRC and the SNP rs12794714 in the vitamin D 25-hydroxylase gene *CYP2R1* (*p* = 0.019), a SNP that has previously been associated with serum vitamin D levels. Two SNPs, rs16847024 in the *GC* gene and rs6022990 in the *CYP24A1* gene, were nominally associated with left-sided CRC (*p* = 0.015 and *p* = 0.018, respectively).

**Conclusions:**

Our results strongly suggest that genetic variation in vitamin D-related genes could affect CRC susceptibility in AAs.

**Electronic supplementary material:**

The online version of this article (doi:10.1007/s10552-014-0361-y) contains supplementary material, which is available to authorized users.

## Introduction

Vitamin D regulates parathyroid hormone levels and has numerous physiological effects, including regulation of bone formation and mineralization, calcium homeostasis, immunity, and insulin secretion [1]. Insufficiency for vitamin D correlates with various diseases, including multiple sclerosis, cardiovascular disease, infectious disease, and cancer [2–6]. In cancer, the vitamin D hypothesis was first proposed based on the observation that cancer mortality varies with higher latitudes [7]. This hypothesis relies on the fact that vitamin D is produced in the skin during exposure to ultraviolet light, and persons living at higher latitudes, being exposed on average to less ultraviolet light compared to persons living at more equatorial latitudes, produce lower levels of vitamin D. Animal studies [8–10] and case–control studies in humans [11–15] have provided strong evidence that vitamin D protects against colorectal cancer (CRC). 25 hydroxyvitamin D_3_ [25(OH)D_3_ or calcidiol] concentrations have been associated with both CRC incidence (op. cit.) and adenoma recurrence [16–18]. Lower dietary intakes of vitamin D have been associated with a higher risk of developing colonic neoplasia [12, 19–21]. Some have suggested that vitamin D insufficiency, which increases with advancing age, is a factor contributing to sporadic CRC, which is also associated with aging [22]. Other factors that are associated with variance in serum 25(OH)D_3_ levels, and coincidentally with CRC risk, include calcium intake, obesity, skin color, and genetic background [23]. Finally, vitamin D has anti-proliferative, anti-invasive, pro-apoptotic, and pro-differentiation activities [24, 25], making vitamin D a potentially potent cancer-preventive agent.

CRC is the second leading cause of cancer-related deaths in both sexes in the United States [26, 27]. Incidence and mortality rates for CRC in the US have declined since the late 1980s, but these trends have been less pronounced in African Americans (AAs), resulting in 20 % higher incidence and 44 % higher mortality rates in AAs compared to European Americans (EAs) [28]. AAs have lower serum vitamin D levels than other Americans [15, 29]. The lower vitamin D levels could be explained in part by skin color, which attenuates the production of vitamin D. Consequently, differences in serum vitamin D levels could contribute to the CRC health disparities between AAs and non-Hispanic whites. Inverse associations between serum vitamin D levels and AA CRC have been reported in the Health Professionals Follow-Up Study and the Multi-Ethnic Cohort [30, 31]; however, the role of vitamin D in protection against CRC in AAs remains understudied.

In the present study, we hypothesized that genetic polymorphisms could affect serum or tissue vitamin D levels and thereby be associated with CRC risk, explaining in part the CRC health disparity in the AA population. We selected genes for analysis that synthesize, metabolize, and transport vitamin D and act in vitamin D-related transcriptional regulation (*CYP2R1*, *CYP3A4*, *CYP24A1*, *CYP27A1*, *CYP27B1*, *GC*, *DHCR7*, and *VDR*, which we will hereafter refer to as vitamin D-related genes). These genes are highly polymorphic in different human populations, and as a group, they have been extensively analyzed in numerous cancer association studies. Although there have been many genetic association studies of CRC [31–35], few studies have focused on the role of vitamin D-related genes in AA CRC [36–38]. Consequently, in the present study, we compared the frequencies of potentially functional single-nucleotide polymorphisms (SNPs) in vitamin D-related genes in AA CRC cases and AA controls.

## Materials and methods

### Human subjects

CRC cases and population-matched, healthy controls were ascertained from the North Carolina Colorectal Cancer Study (NCCCS) and the Chicago Colorectal Cancer Consortium (CCCC). In total, we included DNA samples from 961 AA CRC cases (371 NCCCS, 590 CCCC) and 838 AA controls (380 NCCCS, 458 CCCC). Samples from the NCCCS were obtained through a large-scale, population-based case–control study of colon and rectal cancer, conducted in a 33 county area in central and eastern North Carolina. Histologically confirmed cases were drawn at random from all CRC cases reported to the North Carolina Central Cancer Registry through the rapid ascertainment system. There were two phases of the NCCCS: one from 1996–2000 and one from 2001–2006, in which rectal and rectal–sigmoid cancers were over-sampled. Controls were selected from North Carolina Division of Motor Vehicle lists if under the age of 65, or from a list of Medicare-eligible beneficiaries obtained from the Health Care Financing Administration if over the age of 65. Controls were matched to cases using randomized recruitment strategies, with probabilities based on 5-year age group, sex, and race. The details of this study have been published previously [37, 39].

The CCCC was established to ascertain a significant proportion of CRC cases occurring in Cook Country, Illinois, with IRB approval to enroll CRC patients prospectively, which began in 2011, at six major hospitals in the County (Advocate Christ Medical Center, Jesse Brown Veterans Administration Medical Center, Rush University Medical Center, John H. Stroger Hospital of Cook County, the University of Chicago Medicine, and the University of Illinois Hospital and Health Sciences System). CRC cases are identified in endoscopy, oncology, or surgery clinics and enrolled in the study. Detailed clinical and epidemiological data are collected, and the CCCC preserves and stores specimens of tumor tissue and noninvolved normal colonic mucosa as well as serum, plasma, red blood cells, and DNA from blood.

In the present study, most of the DNA samples from CRC cases (590 individuals) were prepared from noncancerous tissues obtained from colon or rectal surgical specimens (formalin-fixed, paraffin-embedded tissues), archived over the period 1985–2012, ascertained through the records in the Departments of Pathology at the Jesse Brown Veterans Administration Medical Center, John H. Stroger Hospital of Cook County, the University of Chicago Medicine, and the University of Illinois Hospital and Health Sciences System. Individuals known to have hereditary syndromes (familial adenomatous polyposis and Lynch syndrome) or inflammatory bowel disease were excluded. Available baseline characteristics including age, gender, race, colorectal tumor location, histological grade, depth of invasion, nodal involvement, and metastases were recorded. The remaining case DNA samples were prepared from blood specimens obtained from the prospective ascertainment of the CCCC. Control subjects were individuals with tumor-free colon and rectum as determined by colonoscopy or cancer-free individuals as determined by review of their available medical records ascertained through the centralized biobanks of the University of Chicago Medical Center, Department of Medicine, and of the University of Illinois Hospital and Health Sciences System. The age at time of sample collection was used as the age for each control.

Germline DNAs were prepared using Gentra Puregene kits (Qiagen) according to the manufacturer’s instructions. For formalin-fixed, paraffin-embedded tissues, the paraffin was first removed with octane–methanol and the proteinase K extraction step was extended to 3 days, adding fresh enzyme on each day, followed by heating the sample at 95 °C for 15 min prior to protein precipitation.

### Genotyping

The vitamin D-related genes were selected for analysis based on their functions in the synthesis, metabolism, transport, and regulation of vitamin D transcriptional responses. SNPs in the vitamin D-related genes were identified by direct sequencing of PCR products of each exon of each gene in addition to PCR products spanning the 5′ and 3′ untranslated regions and 2,000 base pairs upstream of the transcription start site of each gene. The PCR products were prepared from DNAs from 48 healthy AA persons ascertained at Howard University in Washington, DC, from 2000 to 2005 [38]. SNPs were selected based on the following criteria: The SNP (1) had a minor allele frequency greater than 5 % and (2) was potentially functional, that is, the base pair change was in predicted regulatory sequences in a promoter, 5′ untranslated, or 3′ untranslated region, could affect splicing, or caused an amino acid substitution or (3) was associated with serum D levels in genome-wide association studies [40, 41]. The list of 39 potentially functional SNPs is shown in Table 1. We noted that many of the variants identified by the sequencing of AA men and selected by these criteria had high Fst values comparing African- and European-ancestry populations (Table 1). Fst is a measure of allele frequency differences between populations.

**Table 1:** Characteristics of the single-nucleotide polymorphisms used in the study

SNP	Gene	Chr	Bp	Alleles	MAF (ASW)	MAF (CEU)	Fst	Function^a^
rs116071925	*CYP27A1*	2	219,646,701	CA	0.016	0	0.025	5′ UTR
rs115316390	*GC*	4	72,651,159	GA	0	0	0.03	Intronic
rs1155563	*GC*	4	72,643,488	TC	0.074	0.264	0.07	Ahn et al.; Wang et al.
rs16847024	*GC*	4	72,650,679	CA	0.074	0	0.084	Promoter
rs17467825	*GC*	4	72,605,517	AG	0.107	0.241	0.057	DGV
rs2282679	*GC*	4	72,608,383	TG	0.107	0.241	0.056	Wang et al.
rs2298850	*GC*	4	72,614,267	GC	0.066	0.23	0.075	Wang et al.
rs3733359	*GC*	4	72,649,774	GA	0.221	0.057	0.115	Intronic_S
rs3755967	*GC*	4	72,609,398	CT	0.107	0.241	0.051	Wang et al.
rs7041	*GC*	4	72,618,334	AC	0.156	0.42	0.248	Missense
rs2740574	*CYP3A4*	7	99,382,096	CT	0.344	0.017	0.601	Promoter
rs10741657	*CYP2R1*	11	14,914,878	GA	0.328	0.414	0.035	Promoter
rs114050796	*CYP2R1*	11	14,914,653	CT	0.066	0	0.032	Promoter
rs12794714	*CYP2R1*	11	14,913,575	GA	0.164	0.431	0.118	Synonymous
rs1993116	*CYP2R1*	11	14,910,234	GA	0.32	0.425	0.039	Ahn et al.; Wang et al.
rs2060793	*CYP2R1*	11	14,915,310	GA	0.41	0.408	0.01	Promoter
rs11234027	*DHCR7*	11	71,234,107	GA	0.254	0.195	0.051	Promoter
rs12785878	*DHCR7*	11	71,167,449	GT	0.352	0.276	0.306	Wang et al.
rs12800438	*DHCR7*	11	71,171,003	GA	0.484	0.276	0.172	Wang et al.
rs3794060	*DHCR7*	11	71,187,679	CT	0.328	0.276	0.325	5′ UTR
rs3829251	*DHCR7*	11	71,194,559	GA	0.18	0.178	0.026	Ahn et al.
rs4944957	*DHCR7*	11	71,168,035	GA	0.426	0.276	0.095	Wang et al.
rs4945008	*DHCR7*	11	71,221,248	AG	0.336	0.282	0.322	DGV
rs7944926	*DHCR7*	11	71,165,625	AG	0.352	0.276	0.306	Wang et al.
rs10877012	*CYP27B1*	12	58,162,085	GT	0.085	0	–	Promoter
rs4646537	*CYP27B1*	12	58,157,281	TG	0.082	0.034	0.011	Intronic
rs11568820	*VDR*	12	48,302,545	TC	0.295	0.236	0.409	Promoter
rs11574038	*VDR*	12	48,277,153	CT	0.025	0	0.024	Intronic
rs11574143	*VDR*	12	48,234,917	CT	0.123	0.08	0.022	DGV
rs1544410	*VDR*	12	48,239,835	CT	0.27	0.489	0.023	Intronic
rs1989969	*VDR*	12	48,278,010	GA	0.459	0.356	0.01	Intronic
rs2228570	*VDR*	12	48,272,895	GA	0.156	0.391	0.072	Missense
rs731236	*VDR*	12	48,238,757	AG	0.279	0.489	0.023	Synonymous
rs2248359	*CYP24A1*	20	52,791,518	TC	0.459	0.391	0.078	Promoter
rs2248461	*CYP24A1*	20	52,792,202	AG	0.467	0.362	0.084	Promoter
rs6013897	*CYP24A1*	20	52,742,479	TA	0.287	0.236	0.012	Wang et al.
rs6022990	*CYP24A1*	20	52,775,532	AG	0.098	0	0.07	Missense
rs73913755	*CYP24A1*	20	52,790,194	GA	0.167	0	–	5′ UTR
rs73913757	*CYP24A1*	20	52,790,518	CT	0.139	0	0.112	Promoter

Fst values were calculated from 1,000 genomes data available through SPSmart (http://spsmart.cesga.es/)

Chr, chromosome; Bp, base pair; alleles, the two variants at the locus with the minor allele shown as the second base of the two shown (underlined); MAF (ASW), minor allele frequency in samples of persons with African ancestry from the southwest of the USA; MAF (CEU), minor allele frequency in samples of persons of European ancestry from Utah; Fst, fixation index

^a^Basis of single-nucleotide polymorphism (SNP) selection for the 39 variants in this study. There were two major criteria for selection: (1) a variant previously identified as associated with serum vitamin D levels (or a SNP in linkage disequilibrium with that variant) or (2) a change in a possible regulatory sequence. The SNPs associated with serum vitamin D levels were taken from Ahn et al. [41] and Wang et al. [40], as indicated. The presumed regulatory variants are indicated by their locations within the gene: 5′ UTR, variant located on the 5′ untranslated region; intronic, an intronic change in or near a possible promoter sequence; promoter, a sequence variant located 5′ of a gene; DGV, downstream gene variant, a sequence variant located 3′ of a gene; intronic_S, a variant that occurred within an intron and in a region important for splicing; missense, a variant resulting in amino acid change; synonymous, a variant resulting in no change to the encoded amino acid, selected based on previous association data

We genotyped the 39 potentially functional SNPs and 100 ancestry informative markers (AIMs) [42] using the Sequenom MassARRAY platform. For quality control, we excluded SNPs with Hardy–Weinberg equilibrium *p* values < 0.001 in controls, which is the significance threshold after adjustment for multiple testing. SNPs and individuals with missingness >10 % were also excluded. As a result, 35 SNPs were included for analysis of the study group. Genotyping rates were >98.6 % for all samples. The concordance rate for 32 duplicate samples was 99.9 %.

### Statistical analysis

Global individual ancestry was determined for each individual in the study group using 100 AIMs for West African ancestry (WAA). Individual ancestry estimates were obtained from the genotype data using the Markov Chain Monte Carlo (MCMC) method implemented in the program STRUCTURE 2.1 [43]. STRUCTURE 2.1 assumes an admixture model using prior population information and independent allele frequencies. The MCMC model was run using *K* = 3 populations with genotype data from 58 Europeans, 67 Native Americans, and 62 West Africans. We used a burn-in length of 30,000 iterations followed by 70,000 replications. To test heterogeneity in the two study groups (NCCCS and CCCC), we analyzed WAA using a principal component analysis (PCA) of the 100 AIMs, gender by a two-sided chi-square test, and age by a two-sided *t* test.

We tested the 35 potentially functional SNPs for association with CRC in the combined NCCCS and CCCC study groups and in each study group individually. We calculated odds ratios (ORs) and 95 % confidence intervals (CIs) using logistic regression assuming a log-additive genetic model. For stratified association testing by anatomic site, we defined right-sided CRC (R-CRC) as adenocarcinoma in the colon proximal to the splenic flexure and left-sided CRC (L-CRC) as adenocarcinoma in the colon and rectum distal to and including the splenic flexure. We also performed an analysis of SNP associations with rectal cancer. To adjust for multiple testing, we calculated gene-wide significance levels by permuting case–control status and repeating the analysis 1,000 times to determine the *p* value from the empirical distribution; *p* values less than 0.05 were taken as significant. Logistic regression analyses were carried out using the program Golden Helix (Bozeman, MO) and PLINK (http://pngu.mgh.harvard.edu/~purcell/plink/).

## Results

### Analysis of all AA CRC cases

Table 2 shows the distribution of CRC cases and controls by sex, age, and percent WAA in the NCCCS and CCCC study groups and in the two study groups combined. The two study groups were comparable with respect to WAA by PCA plot (Supplementary Figure 1) and gender (*p* = 0.463). Age was significantly different between the two study groups (*p* < 0.001); however, the age difference was not large (means ages were 63.6 in the NCCCS vs. 61.5 in the CCCC). There was significant heterogeneity within the study groups with respect to age, gender, and ancestry; consequently, we adjusted for these parameters in the logistic regression models.

**Table 2:** Clinical characteristics of the two study groups

	Cases	Controls
*NCCC study*		
Number of subjects	371	380
Mean age and SD	62 ± 10	65 ± 6
Mean %WAA and SD	83 ± 13	82 ± 15
Gender (M/F)	192/179	181/199
*CCCC study*		
Number of subjects	590	458
Mean age and SD	64 ± 13	57 ± 13
Mean %WAA and SD	83 ± 16	86 ± 16
Gender (M/F)	331/259	169/289
*Pooled study*		
Number of subjects	961	838
Mean age and SD	63 ± 12	61 ± 13
Mean %WAA and SD	83 ± 14	84 ± 15
Gender (M/F)	523/438	350/488

Association ORs and *p* values were calculated from comparisons of CRC cases and controls in the combined NCCCS and CCCC study groups, and selected SNPs (*p* < 0.1) are shown in Table 3. ORs and *p* value results for all SNPs in the combined and individual study groups are shown in Supplementary Table 1. After adjustment for age, sex, and WAA, the A allele of SNP rs12794714, located in the 25-hydroxylase gene *CYP2R1*, was associated with a decreased risk of CRC in the combined CRC groups (*p* = 0.019; OR = 0.79, 95 % CI 0.65–0.96). The associations of the minor alleles of two other SNPs—rs17467825 and rs7041—trended toward significance with *p* values between 0.05 and 0.1 (Table 3). rs12794714 was not significantly associated with CRC in either study group alone (Supplementary Table 1); however, this SNP was still significant after adjustment for multiple testing on a gene-wide basis (Adj *p* = 0.048).

**Table 3:** Selected genetic polymorphisms that associate with colorectal cancer in the combined series

SNPs	Gene	MA	MAF	Genotype counts^a^						OR (95 % CI)	*p* value	Adj *p*
				11 Case	11 Cont	12 Case	12 Cont	22 Case	22 Cont			
rs12794714	*CYP2R1*	A	0.183	638 (71)	501 (66)	253 (28)	239 (31)	11 (1.2)	20 (1.4)	0.79 (0.65–0.96)	0.019	0.048
rs7041	*GC*	G	0.196	585 (68)	478 (64)	234 (27)	244 (33)	39 (4.5)	26 (2)	0.84 (0.69–1.01)	0.068	0.321
rs17467825	*GC*	G	0.106	774 (84)	639 (80)	137 (15)	150 (19)	12 (1.3)	8 (0.5)	0.81 (0.64–1.03)	0.079	0.407

SNP, single-nucleotide polymorphism; MA, minor allele; MAF, minor allele frequency; OR odds ratio; CI, confidence interval; *p* value calculated by logistic regression adjusted for age, sex, and West African ancestry, Adj *p*, permutated *p* value (1,000 permutations) adjusted for age, sex, and West African ancestry

^a^Genotype counts for each class—11 homozygous for the major allele, 12 heterozygous, and 22 homozygous for the minor allele—in cases and controls (cont)

### Analysis of AA CRC cases stratified by tumor location

Because cancer on the right and left sides of the colon is different at the molecular level [44], we analyzed R-CRC and L-CRC separately. We compared the 292 R-CRC cases from the combined NCCCS and CCCC study groups with all 838 controls; similarly, we compared the combined 443 L-CRC cases with all 838 controls. Association ORs and *p* values were calculated by logistic regression, and selected SNPs (*p* < 0.1) are shown in Table 4. ORs and *p* values for all SNPS in the combined and individual study groups are shown in Supplementary Table 2. In the analysis of genotype data from R-CRC cases, we obtained results comparable to the analysis of all CRC cases. The A allele of the *CYP2R1* SNP rs12794714 and the G allele of rs7041 in *CYP24A1* were weakly associated with the decreased risk of R-CRC (*p* = 0.056 and *p* = 0.059, respectively) (Table 4). rs12794714 was significantly associated with R-CRC in the NCCCS group but not in the CCCC study group. None of the *p* values adjusted for multiple testing were less than 0.1.

**Table 4:** Selected genetic polymorphisms that associate with left-sided colorectal cancer or right-sided colorectal cancer

SNPs	Gene	MA	MAF	L-CRC			R-CRC		
				OR (95 % CI)	*p* value	Adj *p*	OR (95 % CI)	*p* value	Adj *p*
rs12794714	*CYP2R1*	A	0.183	0.83 (0.65–1.07)	0.15	0.88	0.75 (0.56–1.01)	0.056	0.14
rs7041	*GC*	G	0.196	0.90 (0.71–1.13)	0.35	0.99	0.77 (0.58–1.01)	0.059	0.26
rs16847024	*GC*	T	0.082	1.49 (1.08–2.06)	0.015	0.091	0.92 (0.61–1.37)	0.670	1
rs6022990	*CYP24A1*	G	0.093	1.40 (1.06–1.86)	0.018	0.087	0.94 (0.64–1.37)	0.730	1
rs73913757	*CYP24A1*	T	0.183	0.81 (0.65–1.01)	0.055	0.238	0.97 (0.75–1.27)	0.830	1
rs17467825	*GC*	G	0.106	0.77 (0.58–1.04)	0.085	0.374	0.87 (0.61–1.22)	0.420	1

SNP, single-nucleotide polymorphism; MA, minor allele; MAF, minor allele frequency; OR, odds ratio; CI, confidence interval; *p* value calculated by logistic regression adjusted for age, sex, and West African ancestry, Adj *p*, permutated *p* value (1,000 permutations) adjusted for age, sex, and West African ancestry; L-CRC, left-sided colorectal cancer; R-CRC, right-sided colorectal cancer

In the analysis of genotype data from L-CRC cases, the T allele of rs16847024 in *GC* was associated with an increased risk of L-CRC (*p* = 0.015; OR = 1.49, 95 % CI 1.08–2.06) (Table 4). The G allele of rs6022990 in *CYP24A1* was also associated with an increased risk of L-CRC (*p* = 0.018; OR = 1.41, 95 % CI 1.06–1.86) (Table 4). Two additional SNPs rs17467825 and rs73913757, localized in the *GC* and *CYP24A1* genes, respectively, trended toward significance in L-CRC (Table 4). rs16847024 and rs6022990 were both significantly associated with L-CRC in the NCCCS group but not in the CCCC, and their *p* values adjusted for multiple testing trended toward significance. Neither was significantly associated with R-CRC or all CRC.

We also analyzed the genotype data comparing rectal cancer cases with controls. The *GC* SNP rs16847024 was more strongly associated with rectal cancer than with L-CRC (*p* = 0.002; OR = 2.29, 95 % CI 1.39–3.75), but none of the other SNPs associated with L-CRC or R-CRC were associated with rectal cancer (Supplementary Table 3). There were two additional SNPs with *p* values less than 0.05, but we note that this genotype analysis was based on only 101 rectal cancer cases, and these results should be interpreted cautiously.

## Discussion

In the present study, we identified several nominally significant associations between SNPs in vitamin D-related genes and AA CRC. When testing all CRC cases, we identified an association between SNP rs12794714 in *CYP2R1* and AA CRC. The association between rs12794714 and CRC stayed significant after adjustment for multiple testing gene-wide. In the subgroup analyses by location in the colon, rs12794714 trended toward significance in R-CRC. Two different SNPs, rs16847024 and rs6022990, had nominally significant *p* values in L-CRC but not in R-CRC nor in all CRCs. These associations trended toward significance after adjustment for multiple testing gene-wide. We used a gene-wide adjustment for multiple testing because significant associations with cancer or serum vitamin D levels have been previously reported in vitamin D-related genes and, in particular, for the three genes that showed associations in this study, as detailed below. Consequently, we concluded that genetic variation in the vitamin D-related genes could account for differences in CRC risk among AAs, although replication of these associations in large, independent AA CRC study groups needs to be performed to test these results.

A recent study of the five SNPs (rs2282679, rs10741657, rs12785878, rs11234027, and rs6013897) most strongly associated with serum 25(OH)D_3_ levels, conducted in 13 large cohorts and examining 10,061 CRC cases and 12,768 controls, failed to identify any associations with CRC [36]. We tested all of these SNPs in the present study and similarly failed to identify a significant effect on risk of CRC in AAs. These SNPs account for approximately 5 % of the variance in serum 25(OH)D_3_ levels, which could be too small an effect to impact CRC risk.

The effect of SNPs that affect serum vitamin D levels may be too small to be important in CRC; however, most of the genes studied in the present study are expressed in colonic mucosa and could have effects on the levels of active hormone in the tissues, where vitamin D has its antitumor effects. *CYP27B1* catalyzes a second 1α-hydroxylation step of 25(OH)D_3_ in the kidney and in some extra-renal tissues to produce the active form of vitamin D, 1,25-dihydroxyvitamin D_3_ [1,25(OH)_2_D_3_ or calcitriol]. Cells in the colon and many other tissues, including the prostate, cervix, breast, placenta, pancreas, and brain, express *CYP27B1* mRNA and other 1α-hydroxylase enzymes and thus have the capacity to synthesize 1,25(OH)_2_D_3_ from 25(OH)D_3_ [45–47]. As a consequence of this capacity, the levels of active vitamin D hormone 1,25(OH)_2_D_3_ in colonic tissue could be strongly influenced by serum levels of 25(OH)D_3_.

*CYP2R1* encodes a hepatic microsomal enzyme—one of the enzymes catalyzing 25-hydroxylation of vitamin D in the liver, but it is also expressed in the colon, where it could convert cholecalciferol to 25(OH)D_3_. The SNP that we found associated with all CRC, rs12794714, was previously found to associate with serum 25(OH)D_3_ concentrations [40]. The SNP is present in a promoter and DNAaseI hypersensitive region. The protective effect of the SNP could be explained if it increased transcription of the *CYP2R1* gene, increasing enzyme levels and producing more 25(OH)D_3_ in colon tissue, where it could result in increased active hormone and a lower risk of CRC.

Almost all the 25(OH)D_3_ and active hormone 1,25(OH)_2_D_3_ exist in the circulation bound to serum vitamin D-binding protein (VDBP; also referred to as Gc-globulin), which is encoded by the gene group-specific component (*GC*) [48, 49]. One important function of VDBP is to regulate the half-life of 25(OH)D_3_ in the circulation through stabilization of the hormone, but VDBP also helps maintain serum vitamin D levels through reuptake by proximal tubule cells in the kidney [50]. 25(OH)D_3_ and 1,25(OH)_2_D_3_ can enter cells either by diffusion of free vitamin D across the cell membrane or by megalin-receptor-mediated endocytosis of vitamin D-VDBP complex [51]. *GC* is highly polymorphic with more than 120 known variants and with different frequency distributions in diverse populations. Some SNPs have been previously associated with plasma concentrations of 25(OH)D_3_ [52, 53] and with breast cancer [54, 55]. Here, we found a nominally significant association between risk of L-CRC in AAs and rs16847024 in *GC*. The rs16847024 SNP was selected for study because it is near the promoter of *GC*; however, it is not located within any regulatory elements, and formally rs16847024 may be in linkage disequilibrium with another SNP that impacts *GC* function. Theoretically, lower levels of VDBP or reduced function could result is less 25(OH)D_3_ delivery to tissues. We note that rs16847024 has not been associated with AA prostate cancer [38] or with vitamin D levels in AAs [56]. rs16847024 is relatively common in African-ancestry populations (minor allele frequency = 0.074), but is not present in European-ancestry populations (Table 1), making this association African ancestry specific.

Although the SNP rs7041 exhibited only a trend toward association with AA CRC in our data, this SNP warrants greater scrutiny because recent work on the relationship between VDBP and serum 25(OH)D_3_ levels has suggested that this polymorphism regulates the bioavailability of 25(OH)D_3_ [57, 58]. rs7041 encodes the electrophoretically distinguishable protein isoforms Gc1F common in Africans and Gc1S common in Europeans; Gc1F binds less 25(OH)D_3_ and is associated with lower serum 25(OH)D_3_ levels, resulting by the authors’ calculation in more bioavailable vitamin D [58]. In our data, the minor allele of rs7041 was associated with protection against CRC, consistent with the usual theory that higher serum 25(OH)D_3_ levels lead to more antitumor activity at the level of the tissues.

*CYP24A1* begins the catabolic processing of 1,25(OH)_2_D_3_, and it may also catabolize 25(OH)D_3_, a process that has the potential to limit the availability of this molecule [59]. Some genetic polymorphisms in *CYP24A1* have been associated with the concentrations of serum vitamin D metabolites [60] and with risk of CRC [32] in European-ancestry populations. Here, we found an association between risk of L-CRC and rs6022990—another SNP that is relatively common in African-ancestry populations (minor allele frequency = 0.098) but not present in European-ancestry populations (Table 1). rs6022990 is a missense variant that substitutes a threonine for a methionine at amino acid residue 374 in CYP24A1. The change is predicted to damage the function of the enzyme by both Polyphen and SIFT, and analysis of over-expressed mutant protein in colon cancer cells HCT116 demonstrated decreased CYP24A1 and increased intracellular levels of 1,25(OH)_2_D_3_ [61]. Based on the usual theory, these experimental results predict that the threonine-encoding allele of rs6022990 would be associated with lower risk of CRC because more active hormone in the colonic mucosa should result in greater antitumor activity. Our results were not consistent with this prediction. Additional AA CRC association studies and direct measurements of CYP24A1 activity in different genetic backgrounds would be useful to test this apparent conflict.

Associations between SNPs and development of CRC by location in the colon have been noted in several studies [62–64]. Biological differences by location in the colon could explain why a SNP has an effect on risk in one part of the colon but not the other part. For instance, the arterial supply for the right and left intestine is different, the lymphatic drainage into colic nodes vary by location, the mucosa is thinner on the right than on the left, and luminal contents differ [65–67]. Clinically, a higher proportion of CRCs in the right colon exhibit lymphocytic infiltrations compared with the left, and on average, R-CRC has a worse prognosis compared to L-CRC [68–72]. AAs have a higher rate of R-CRC development compared to EAs [73], and consistent with this observation, AAs have a higher proportion of CRCs with lymphocytic infiltrations [63]. There is evidence that the vitamin D-related genes *VDR* in human and *Cyp24a1* and *Cyp27b1* in mouse are regulated differently in the proximal and distal colon [74, 75], raising the question whether vitamin D levels could affect CRC development and outcomes by location within the colon [76].

An important limitation of this study is the lack of data on serum vitamin D levels from cases and controls that would allow us to test our central hypothesis more directly. Our study was also limited by lack of information about important covariates that modify vitamin D levels. Sunlight exposure strongly influences serum vitamin D levels; only about a quarter of the interindividual variability in serum vitamin D concentration is attributable to season, geographical latitude, or vitamin D intake [77]. Variation in serum vitamin D levels is modified by calcium intake, age, obesity, skin pigmentation, physical activity, race, and genetic background [36]. Further studies in AA study groups in which serum 25(OH)D_3_ levels and additional epidemiological data are available are needed to control for these effects. Although we have some mechanistic clues about the possible functions of the polymorphisms that exhibited associations in this study, the in vitro information is relatively limited and studies in human tissues have not been performed. None of the associated SNPs have been reported as cis-expression quantitative trait loci (cis-eQTL) either in publicly available databases (http://www.scandb.org) or in our own unpublished eQTL study of AA colon tissue (data not shown). Finally, although this study contains the largest number of AAs genotyped for these vitamin D-related SNPs, larger study groups are needed to test the validity of the associations we report here and to increase overall study power.

Although the exact molecular mechanisms by which the SNPs in *CYP2R1, CYP24A1*, and *GC* influence CRC development remain to be determined, our study provides evidence that SNPs in vitamin D-related genes play a role in CRC susceptibility in AAs. Our findings are significant because there has been limited focus on the role of vitamin D-related polymorphisms on CRC in AAs. Identifying genetic variants affecting the functional status of vitamin D-related genes is important for understanding the role of this important regulatory hormone in tumor development and progression.

## Electronic supplementary material

Below is the link to the electronic supplementary material.

Supplementary material 1 (DOCX 46 kb)

Supplementary material 2 (DOCX 480 kb)
