# Supplementary material for: Genetic variation in vitamin D-related genes and risk of colorectal cancer in African Americans
Source: Cancer Causes Control. 2014 Feb 23;25(5):561–70. doi: 10.1007/s10552-014-0361-y (PMC3978221; doi:10.1007/s10552-014-0361-y)
Supplement: Supplementary file 1 — Supplementary material 1 (DOCX 46 kb) [file 10552_2014_361_MOESM1_ESM.docx]

**Supplementary Table 1**. Odds ratios and *P* values of comparisons of African American colorectal cancer cases and controls for polymorphisms in the vitamin D pathway genes, calculated in the combined study groups and in each separate study group

|  | | **Total CRC^1^** | | | | | **NCCC CRC^1^** | | **CCCC CRC^1^** | |
| --- | --- | --- | --- | --- | --- | --- | --- | --- | --- | --- |
| **SNP** | **Gene** | **Fcas** | **Fcont** | **unadjusted OR (95% CI)** | **OR (95% CI)** | ***P*** | **OR (95% CI)** | ***P*** | **OR (95% CI)** | ***P*** |
| rs116071925 | *CYP27A1* | 0.015 | 0.021 | 0.72 (0.43–1.21) | 0.77 (0.45-1.33) | 0.346 | 0.99 (0.68-1.46) | 0.195 | 0.98 (0.46-2.11) | 0.967 |
| rs115316390 | *GC* | 0.009 | 0.005 | 1.62 (0.68–3.85) | 1.51 (0.62-3.67) | 0.360 | 2.27 (0.55-9.44) | 0.244 | 1.21 (0.37-3.99) | 0.750 |
| rs1155563 | *GC* | 0.096 | 0.115 | 0.82 (0.66–1.03) | 0.83 (0.66-1.05) | 0.122 | 0.80 (0.57-1.13) | 0.201 | 0.80 (0.58-1.12) | 0.192 |
| rs16847024 | *GC* | 0.082 | 0.069 | 1.22 (0.94–1.59) | 1.21 (0.92-1.60) | 0.176 | 1.51 (0.99-2.31) | 0.054 | 0.98 (0.67-1.44) | 0.907 |
| rs17467825 | *GC* | 0.087 | 0.106 | 0.81 (0.64–1.02) | 0.81 (0.64-1.03) | 0.079 | 0.76 (0.53-1.10) | 0.142 | 0.82 (0.58-1.15) | 0.245 |
| rs2282679 | *GC* | 0.080 | 0.092 | 0.86 (0.68–1.1) | 0.86 (0.67-1.11) | 0.241 | 0.77 (0.53-1.12) | 0.175 | 0.92 (0.64-1.34) | 0.673 |
| rs2298850 | *GC* | 0.067 | 0.081 | 0.83 (0.64–1.08) | 0.84 (0.64-1.10) | 0.210 | 0.82 (0.55-1.21) | 0.313 | 0.87 (0.59-1.29) | 0.480 |
| rs3733359 | *GC* | 0.220 | 0.223 | 0.99 (0.84–1.16) | 0.98 (0.82-1.16) | 0.780 | 0.99 (0.77-1.29) | 0.968 | 0.93 (0.73-1.18) | 0.540 |
| rs3755967 | *GC* | 0.085 | 0.095 | 0.89 (0.7–1.13) | 0.89 (0.69-1.14) | 0.354 | 0.76 (0.53-1.11) | 0.154 | 0.97 (0.68-1.39) | 0.861 |
| rs7041 | *GC* | 0.183 | 0.196 | 0.92 (0.77–1.09) | 0.84 (0.69-1.01) | 0.068 | 0.80 (0.60-1.07) | 0.131 | 0.93 (0.71-1.21) | 0.580 |
| rs2740574 | *CYP3A4* | 0.380 | 0.366 | 1.06 (0.92–1.22) | 1.00 (0.86-1.17) | 0.973 | 1.09 (0.87-1.36) | 0.453 | 1.00 (0.80-1.24) | 0.965 |
| rs10741657 | *CYP2R1* | 0.258 | 0.273 | 0.93 (0.79–1.08) | 0.92 (0.78-1.08) | 0.312 | 0.86 (0.68-1.10) | 0.232 | 1.01 (0.80-1.28) | 0.909 |
| rs114050796 | *CYP2R1* | 0.051 | 0.053 | 0.96 (0.7–1.32) | 1.05 (0.75-1.47) | 0.769 | 1.32 (0.79-2.20) | 0.283 | 0.86 (0.54-1.40) | 0.563 |
| rs12794714 | *CYP2R1* | 0.149 | 0.183 | 0.77 (0.63–0.93) | 0.79 (0.65-0.96) | 0.019 | 0.75 (0.55-1.03) | 0.070 | 0.80 (0.60-1.06) | 0.116 |
| rs1993116 | *CYP2R1* | 0.264 | 0.278 | 0.93 (0.79–1.08) | 0.93 (0.79-1.09) | 0.366 | 0.84 (0.66-1.07) | 0.152 | 1.04 (0.82-1.32) | 0.742 |
| rs2060793 | *CYP2R1* | 0.342 | 0.362 | 0.92 (0.8–1.06) | 0.92 (0.80-1.08) | 0.310 | 0.89 (0.71-1.12) | 0.330 | 0.99 (0.80-1.23) | 0.946 |
| rs11234027 | *DHCR7* | 0.329 | 0.311 | 1.08 (0.93–1.26) | 1.11 (0.95-1.30) | 0.196 | 1.15 (0.91-1.45) | 0.251 | 1.06 (0.85-1.33) | 0.600 |
| rs12785878 | *DHCR7* | 0.253 | 0.271 | 0.91 (0.78–1.06) | 0.88 (0.74-1.04) | 0.122 | 0.58 (0.25-1.35) | 0.983 | 0.80 (0.63-1.02) | 0.075 |
| rs12800438 | *DHCR7* | 0.392 | 0.389 | 1.01 (0.88–1.16) | 1.02 (0.88-1.18) | 0.825 | 1.11 (0.89-1.37) | 0.365 | 0.96 (0.78-1.19) | 0.728 |
| rs3794060 | *DHCR7* | 0.234 | 0.241 | 0.96 (0.82–1.13) | 0.92 (0.77-1.10) | 0.358 | 1.00 (0.77-1.31) | 0.988 | 0.86 (0.68-1.10) | 0.224 |
| rs3829251 | *DHCR7* | 0.275 | 0.276 | 1.00 (0.84–1.18) | 1.00 (0.83-1.19) | 0.970 | 1.03 (0.85-1.21) | 0.347 | 0.89 (0.70-1.12) | 0.319 |
| rs4944957 | *DHCR7* | 0.483 | 0.489 | 0.98 (0.85–1.12) | 0.96 (0.83-1.10) | 0.547 | 0.91 (0.74-1.12) | 0.382 | 0.94 (0.77-1.17) | 0.595 |
| rs4945008 | *DHCR7* | 0.230 | 0.239 | 0.95 (0.81–1.12) | 0.91 (0.77-1.08) | 0.296 | 1.04 (0.79-1.36) | 0.785 | 0.84 (0.66-1.07) | 0.158 |
| rs7944926 | *DHCR7* | 0.250 | 0.256 | 0.97 (0.82–1.14) | 0.94 (0.89-1.12) | 0.479 | 1.14 (0.87-1.51) | 0.340 | 0.80 (0.63-1.02) | 0.074 |
| rs10877012 | *CYP27B1* | 0.138 | 0.118 | 1.19 (0.96–1.46) | 1.15 (0.93-1.43) | 0.187 | 1.01 (0.73-1.40) | 0.939 | 1.12 (0.83-1.51) | 0.463 |
| rs4646537 | *CYP27B1* | 0.089 | 0.090 | 1.00 (0.78–1.27) | 1.02 (0.79-1.32) | 0.859 | 1.18 (0.81-1.70) | 0.392 | 0.96 (0.66-1.38) | 0.811 |
| rs11574038 | *VDR* | 0.038 | 0.034 | 1.11 (0.77–1.61) | 1.17 (0.79-1.73) | 0.431 | 1.08 (0.83-1.40) | 0.099 | 0.78 (0.45-1.37) | 0.386 |
| rs11574143 | *VDR* | 0.082 | 0.086 | 0.96 (0.75–1.22) | 0.90 (0.70-1.16) | 0.424 | 1.62 (0.91-2.91) | 0.976 | 0.76 (0.53-1.10) | 0.143 |
| rs1544410 | *VDR* | 0.290 | 0.295 | 0.97 (0.83–1.14) | 1.00 (0.84-1.19) | 0.997 | 1.13 (0.88-1.44) | 0.341 | 0.92 (0.71-1.18) | 0.488 |
| rs731236 | *VDR* | 0.276 | 0.274 | 1.01 (0.86–1.19) | 1.05 (0.88-1.25) | 0.599 | 0.97 (0.73-1.28) | 0.810 | 1.10 (0.87-1.40) | 0.420 |
| rs2248359 | *CYP24A1* | 0.352 | 0.350 | 1.01 (0.87–1.16) | 1.01 (0.87-1.17) | 0.910 | 1.09 (0.87-1.37) | 0.451 | 0.95 (0.77-1.18) | 0.651 |
| rs6013897 | *CYP24A1* | 0.238 | 0.246 | 0.96 (0.82–1.12) | 0.98 (0.83-1.15) | 0.782 | 0.96 (0.75-1.23) | 0.758 | 0.01 (0.80-1.29) | 0.924 |
| rs6022990 | *CYP24A1* | 0.093 | 0.083 | 1.13 (0.89–1.43) | 1.15 (0.89-1.47) | 0.282 | 1.48 (1.00-2.19) | 0.046 | 1.04 (0.73-1.48) | 0.830 |
| rs73913755 | *CYP24A1* | 0.161 | 0.175 | 0.91 (0.76–1.09) | 0.91 (0.75-1.11) | 0.3495 | 0.93 (0.70-1.24) | 0.611 | 0.85 (0.64-1.12) | 0.244 |
| rs73913757 | *CYP24A1* | 0.183 | 0.203 | 0.88 (0.73–1.05) | 0.90 (0.74-1.08) | 0.252 | 1.01 (0.77-1.33) | 0.938 | 0.83 (0.63-1.10) | 0.191 |

^1^Total CRC, calculations in the combined study groups; NCCCS CRC, calculations in the North Carolina Colorectal Cancer Study group; CCCC CRC, calculations in the Chicago Colorectal Cancer study group. The *P* values were calculated by logistic regression adjusted for sex, age, and West African ancestry.

SNP, single nucleotide polymorphism; Fcas, frequency of the minor allele in cases; Fcont, frequency of the minor allele in controls; OR, odds ratio; CI, confidence interval; unadjusted OR, odds ratios calculated by logistic regression with no adjustments; OR, odds ratios calculated by logistic regression adjusted for sex, age, and West African ancestry.

**Supplementary Table 2**. Odds ratios and *P* values of comparisons of African American colorectal cancer cases and controls for polymorphisms in the vitamin D pathway genes, calculated in left-sided and right-sided colorectal cancer in the combined study group and in each study groups

|  |  |  | **Odds ratios (95% confidence intervals)** | | | | | | ***P* values** | | | | | |
| --- | --- | --- | --- | --- | --- | --- | --- | --- | --- | --- | --- | --- | --- | --- |
| **SNP_ID** | **Gene** | **Chr** | **Total**  **L-CRC** | **NCCCS**  **L-CRC** | **CCCC**  **L-CRC** | **Total**  **R-CRC** | **NCCCS**  **R-CRC** | **CCCC**  **R-CRC** | **Total**  **L-CRC** | **NCCCS**  **L-CRC** | **CCCC L-CRC** | **Total**  **R-CRC** | **NCCCS**  **R-CRC** | **CCCC R-CRC** |
| rs116071925 | *CYP27A1* | 2 | 0.58 (0.27–1.23) | 0.38 (0.12–1.19) | 0.78 (0.27–2.21) | 1.02 (0.49–2.13) | 0.62 (0.18–2.21) | 1.29 (0.48–3.47) | 0.135 | 0.074 | 0.630 | 0.962 | 0.444 | 0.624 |
| rs115316390 | *GC* | 4 | 1.34 (0.46–3.94) | 2.41 (0.5–11.53) | 0.94 (0.17–5.17) | 1.94 (0.65–5.78) | 2.74 (0.45–16.82) | 1.71 (0.4–7.25) | 0.598 | 0.269 | 0.945 | 0.245 | 0.300 | 0.471 |
| rs16847024 | *GC* | 4 | 1.49 (1.08–2.06) | 1.75 (1.1–2.79) | 1.31 (0.82–2.07) | 0.92 (0.61–1.37) | 0.99 (0.5–1.96) | 0.8 (0.48–1.36) | 0.015 | 0.018 | 0.259 | 0.665 | 0.976 | 0.407 |
| rs17467825 | *GC* | 4 | 0.77 (0.58–1.04) | 0.67 (0.43–1.03) | 0.84 (0.54–1.31) | 0.87 (0.61–1.22) | 0.83 (0.49–1.43) | 0.84 (0.52–1.33) | 0.085 | 0.064 | 0.438 | 0.422 | 0.503 | 0.447 |
| rs2282679 | *GC* | 4 | 0.81 (0.59–1.12) | 0.68 (0.44–1.06) | 0.94 (0.58–1.53) | 0.92 (0.65–1.32) | 0.81 (0.46–1.42) | 0.97 (0.59–1.58) | 0.196 | 0.083 | 0.799 | 0.658 | 0.451 | 0.906 |
| rs2298850 | *GC* | 4 | 0.84 (0.6–1.17) | 0.83 (0.53–1.3) | 0.78 (0.46–1.32) | 0.82 (0.55–1.22) | 0.68 (0.36–1.29) | 0.94 (0.55–1.6) | 0.305 | 0.414 | 0.350 | 0.329 | 0.222 | 0.806 |
| rs3733359 | *GC* | 4 | 1.00 (0.82–1.23) | 1.02 (0.76–1.36) | 0.97 (0.72–1.31) | 1.06 (0.84–1.35) | 1.08 (0.75–1.56) | 1.03 (0.74–1.42) | 0.973 | 0.909 | 0.850 | 0.601 | 0.693 | 0.875 |
| rs3755967 | *GC* | 4 | 0.86 (0.63–1.17) | 0.69 (0.45–1.08) | 1.02 (0.64–1.62) | 0.91 (0.63–1.3) | 0.74 (0.42–1.33) | 0.97 (0.6–1.57) | 0.324 | 0.097 | 0.936 | 0.587 | 0.306 | 0.914 |
| rs7041 | *GC* | 4 | 0.9 (0.71–1.13) | 0.85 (0.61–1.18) | 1.01 (0.72–1.41) | 0.77 (0.58–1.01) | 0.73 (0.47–1.15) | 0.87 (0.59–1.26) | 0.351 | 0.323 | 0.973 | 0.059 | 0.169 | 0.451 |
| rs2740574 | *CYP3A4* | 7 | 1.03 (0.86–1.24) | 1.15 (0.89–1.48) | 0.96 (0.73–1.27) | 0.99 (0.8–1.23) | 0.94 (0.67–1.32) | 1.1 (0.81–1.49) | 0.749 | 0.278 | 0.779 | 0.952 | 0.728 | 0.539 |
| rs10741657 | *CYP2R1* | 11 | 0.92 (0.76–1.12) | 0.85 (0.65–1.12) | 1.04 (0.77–1.40) | 1 (0.8–1.26) | 0.93 (0.66–1.32) | 1.12 (0.81–1.54) | 0.416 | 0.256 | 0.813 | 0.973 | 0.683 | 0.498 |
| rs114050796 | *CYP2R1* | 11 | 1.27 (0.86–1.86) | 1.37 (0.78–2.43) | 1.21 (0.7–2.10) | 0.61 (0.35–1.08) | 0.94 (0.42–2.12) | 0.45 (0.2–1.03) | 0.230 | 0.280 | 0.505 | 0.074 | 0.879 | 0.042 |
| rs12794714 | *CYP2R1* | 11 | 0.83 (0.65–1.07) | 0.88 (0.61–1.27) | 0.83 (0.58–1.20) | 0.75 (0.56–1.01) | 0.80 (0.58–1.31) | 0.88 (0.6–1.29) | 0.147 | 0.337 | 0.321 | 0.056 | 0.011 | 0.495 |
| rs1993116 | *CYP2R1* | 11 | 0.96 (0.79–1.07) | 0.85 (0.65–1.11) | 1.13 (0.84–1.52) | 0.97 (0.77–1.22) | 0.86 (0.61–1.23) | 1.13 (0.81–1.57) | 0.714 | 0.233 | 0.433 | 0.779 | 0.409 | 0.470 |
| rs2060793 | *CYP2R1* | 11 | 0.95 (0.79–1.14) | 0.89 (0.69–1.15) | 1.05 (0.8–1.39) | 0.92 (0.74–1.14) | 0.91 (0.66–1.27) | 0.96 (0.72–1.3) | 0.575 | 0.357 | 0.709 | 0.430 | 0.586 | 0.813 |
| rs11234027 | *DHCR7* | 11 | 1.14 (0.95–1.38) | 1.09 (0.83–1.43) | 1.21 (0.91–1.61) | 1.19 (0.95–1.48) | 1.45 (1.03–2.04) | 0.99 (0.73–1.35) | 0.166 | 0.526 | 0.183 | 0.123 | 0.033 | 0.966 |
| rs12785878 | *DHCR7* | 11 | 0.89 (0.73–1.1) | 0.93 (0.7–1.24) | 0.87 (0.64–1.18) | 0.94 (0.74–1.19) | 1.08 (0.75–1.58) | 0.86 (0.62–1.2) | 0.285 | 0.625 | 0.370 | 0.587 | 0.671 | 0.383 |
| rs12800438 | *DHCR7* | 11 | 1.11 (0.93–1.32) | 1.1 (0.86–1.41) | 1.12 (0.86–1.45) | 0.99 (0.8–1.23) | 1.17 (0.84–1.63) | 0.87 (0.65–1.16) | 0.255 | 0.433 | 0.416 | 0.951 | 0.359 | 0.336 |
| rs3794060 | *DHCR7* | 11 | 0.94 (0.76–1.16) | 0.94 (0.69–1.27) | 0.93 (0.68–1.26) | 1.03 (0.81–1.31) | 1.17 (0.79–1.74) | 0.93 (0.68–1.29) | 0.580 | 0.689 | 0.627 | 0.829 | 0.423 | 0.684 |
| rs3829251 | *DHCR7* | 11 | 0.98 (0.79–1.22) | (–) | 1.00 (0.74–1.35) | 1.21 (0.95–1.55) | (–) | 0.94 (0.69–1.28) | 0.860 | (–) | 0.990 | 0.129 | (–) | 0.690 |
| rs4944957 | *DHCR7* | 11 | 0.91 (0.76–1.08) | 0.91 (0.72–1.16) | 0.84 (0.64–1.11) | 0.96 (0.78–1.17) | 0.88 (0.65–1.2) | 1.02 (0.76–1.36) | 0.274 | 0.457 | 0.218 | 0.668 | 0.409 | 0.906 |
| rs4945008 | *DHCR7* | 11 | 0.92 (0.75–1.14) | 0.95 (0.7–1.29) | 0.91 (0.68–1.24) | 1.03 (0.81–1.31) | 1.27 (0.86–1.89) | 0.92 (0.66–1.27) | 0.448 | 0.759 | 0.563 | 0.821 | 0.234 | 0.600 |
| rs7944926 | *DHCR7* | 11 | 0.97 (0.78–1.2) | 1.1 (0.8–1.5) | 0.87 (0.64–1.18) | 1.01 (0.78–1.3) | 1.2 (0.78–1.86) | 0.89 (0.64–1.23) | 0.760 | 0.574 | 0.371 | 0.957 | 0.406 | 0.469 |
| rs10877012 | *CYP27B1* | 12 | 1.22 (0.94–1.57) | 1.03 (0.71–1.49) | 1.22 (0.84–1.78) | 1.07 (0.79–1.45) | 0.96 (0.58–1.58) | 1.02 (0.69–1.52) | 0.139 | 0.891 | 0.293 | 0.645 | 0.875 | 0.911 |
| rs4646537 | *CYP27B1* | 12 | 1.01 (0.74–1.37) | 1.19 (0.78–1.81) | 0.89 (0.56–1.43) | 1.17 (0.83–1.66) | 1.36 (0.8–2.31) | 1.2 (0.74–1.94) | 0.954 | 0.428 | 0.633 | 0.366 | 0.262 | 0.473 |
| rs1155563 | *VDR* | 12 | 0.84 (0.63–1.11) | 0.74 (0.49–1.1) | 0.86 (0.56–1.30) | 0.82 (0.58–1.15) | 0.75 (0.44–1.27) | 0.81 (0.51–1.29) | 0.215 | 0.134 | 0.468 | 0.248 | 0.278 | 0.362 |
| rs11574038 | *VDR* | 12 | 1.2 (0.76–1.92) | 1.57 (0.83–2.99) | 0.75 (0.35–1.60) | 1.19 (0.68–2.09) | 2.02 (0.92–4.41) | 0.71 (0.31–1.64) | 0.439 | 0.166 | 0.445 | 0.538 | 0.088 | 0.408 |
| rs11574143 | *VDR* | 12 | 0.97 (0.71–1.31) | 0.91 (0.59–1.42) | 0.92 (0.6–1.41) | 0.95 (0.67–1.36) | 1.24 (0.73–2.09) | 0.71 (0.43–1.18) | 0.821 | 0.685 | 0.700 | 0.778 | 0.436 | 0.175 |
| rs1544410 | *VDR* | 12 | 1.08 (0.89–1.32) | 1.29 (0.97–1.7) | 0.85 (0.62–1.16) | 0.9 (0.71–1.15) | 0.89 (0.61–1.29) | 0.91 (0.65–1.27) | 0.441 | 0.076 | 0.304 | 0.397 | 0.528 | 0.571 |
| rs731236 | *VDR* | 12 | 0.97 (0.78–1.21) | 1.07 (0.77–1.47) | 0.88 (0.65–1.21) | 1.19 (0.94–1.52) | 0.88 (0.57–1.34) | 1.35 (0.99–1.84) | 0.798 | 0.693 | 0.436 | 0.153 | 0.545 | 0.061 |
| rs2248359 | *CYP24A1* | 20 | 1.04 (0.86–1.25) | 1.27 (0.97–1.65) | 0.90 (0.69–1.18) | 0.96 (0.78–1.19) | 0.94 (0.67–1.32) | 0.95 (0.71–1.27) | 0.688 | 0.082 | 0.440 | 0.721 | 0.717 | 0.714 |
| rs6013897 | *CYP24A1* | 20 | 0.92 (0.75–1.13) | 0.9 (0.68–1.21) | 0.98 (0.73–1.32) | 1.07 (0.85–1.35) | 1.12 (0.79–1.59) | 1.03 (0.75–1.43) | 0.442 | 0.486 | 0.903 | 0.545 | 0.516 | 0.847 |
| rs6022990 | *CYP24A1* | 20 | 1.40 (1.06–1.86) | 1.75 (1.14–2.68) | 1.31 (0.87–1.95) | 0.94 (0.64–1.37) | 1.16 (0.64–2.13) | 0.95 (0.57–1.6) | 0.018 | 0.010 | 0.196 | 0.730 | 0.626 | 0.858 |
| rs73913755 | *CYP24A1* | 20 | 0.85 (0.67–1.08) | 0.88 (0.64–1.22) | 0.73 (0.51–1.06) | 0.93 (0.71–1.23) | 0.95 (0.63–1.43) | 0.9 (0.61–1.32) | 0.1768 | 0.4546 | 0.091 | 0.622 | 0.809 | 0.588 |
| rs73913757 | *CYP24A1* | 20 | 0.81 (0.65–1.01) | 0.79 (0.58–1.08) | 0.76 (0.53–1.09) | 0.97 (0.75–1.27) | 1.35 (0.91–2) | 0.86 (0.58–1.26) | 0.055 | 0.143 | 0.129 | 0.834 | 0.137 | 0.434 |

SNP, single nucleotide polymorphism; Chr, chromosome; L-CRC, left-sided colorectal cancer; R-CRC, right sided colorectal cancer; Total, the combined study groups; NCCC, North Carolina Colorectal Cancer Study; CCCC Chicago Colorectal Cancer Consortium.

Supplementary Table 3. Odds ratios and *P* values of comparisons of African American rectal cancer cases and controls for polymorphisms in the vitamin D pathway genes, calculated in the combined study group

| **SNPs** | **Gene** | **OR (95% CI)** | ***P* value** | **Adj *P*** |
| --- | --- | --- | --- | --- |
| rs116071925 | *CYP27A1* | 1.15 (0.40-3.32) | 0.801 | 1 |
| rs1155563 | *GC* | 0.59 (0.33-1.05) | 0.053 | 0.767 |
| rs16847024 | *GC* | 2.29 (1.39-3.75) | 0.002 | 0.051 |
| rs17467825 | *GC* | 0.61 (0.33-1.11) | 0.088 | 0.912 |
| rs2282679 | *GC* | 0.54 (0.28-1.06) | 0.056 | 0.783 |
| rs2298850 | *GC* | 0.68 (0.35-1.29) | 0.217 | 0.999 |
| rs3733359 | *GC* | 1.14 (0.80-1.62) | 0.473 | 1 |
| rs3755967 | *GC* | 0.62 (0.33-1.16) | 0.113 | 0.959 |
| rs7041 | *GC* | 0.96 (0.65-1.43) | 0.842 | 1 |
| rs2740574 | *CYP3A4* | 1.01 (0.73-1.40) | 0.944 | 1 |
| rs10741657 | *CYP2R1* | 0.84 (0.58-1.20) | 0.329 | 1 |
| rs114050796 | *CYP2R1* | 0.65 (0.28-1.55) | 0.309 | 1 |
| rs12794714 | *CYP2R1* | 0.92 (0.60-1.42) | 0.719 | 1 |
| rs1993116 | *CYP2R1* | 0.86 (0.60-1.23) | 0.400 | 1 |
| rs2060793 | *CYP2R1* | 0.75 (0.53-1.05) | 0.088 | 0.915 |
| rs11234027 | *DHCR7* | 1.51 (1.08-2.11) | 0.016 | 0.338 |
| rs12785878 | *DHCR7* | 0.77 (0.53-1.13) | 0.177 | 0.995 |
| rs12800438 | *DHCR7* | 1.20 (0.88-1.64) | 0.242 | 1 |
| rs3794060 | *DHCR7* | 0.90 (0.62-1.32) | 0.601 | 1 |
| rs4944957 | *DHCR7* | 0.89 (0.66-1.21) | 0.450 | 1 |
| rs4945008 | *DHCR7* | 0.87 (0.59-1.27) | 0.461 | 1 |
| rs7944926 | *DHCR7* | 0.85 (0.57-1.27) | 0.429 | 1 |
| rs10877012 | *CYP27B1* | 1.63 (1.07-2.48) | 0.026 | 0.493 |
| rs4646537 | *CYP27B1* | 0.55 (0.28-1.08) | 0.064 | 0.815 |
| rs11568820 | *VDR* | 1.15 (0.78-1.70) | 0.475 | 1 |
| rs11574038 | *VDR* | 2.00 (0.98-4.05) | 0.070 | 0.844 |
| rs11574143 | *VDR* | 0.95 (0.56-1.61) | 0.852 | 1 |
| rs1544410 | *VDR* | 1.18 (0.84-1.68) | 0.341 | 1 |
| rs731236 | *VDR* | 1.26 (0.87-1.81) | 0.226 | 0.999 |
| rs2248359 | *CYP24A1* | 1.19 (0.86-1.64) | 0.291 | 1 |
| rs6013897 | *CYP24A1* | 1.24 (0.89-1.73) | 0.201 | 0.998 |
| rs6022990 | *CYP24A1* | 1.37 (0.83-2.25) | 0.236 | 1 |
| rs73913755 | *CYP24A1* | 0.93 (0.61-1.41) | 0.717 | 1 |
| rs73913757 | *CYP24A1* | 0.74 (0.48-1.13) | 0.153 | 0.986 |

SNP, single nucleotide polymorphism; OR, odds ratio; CI, confidence interval; *P* value, calculated by logistic regression and adjusted for age, sex, and West African ancestry; Adj *P*, permutated *P* value (1,000 permutations) adjusted for age, sex, and West African ancestry.
