# Supplementary material for: Genetic variation in vitamin D-related genes and risk of colorectal cancer in African Americans
Source: Cancer Causes Control. 2014 Feb 23;25(5):561–70. doi: 10.1007/s10552-014-0361-y (PMC3978221; doi:10.1007/s10552-014-0361-y)
Supplement: Supplementary file 2 — Supplementary material 2 (DOCX 480 kb) [file 10552_2014_361_MOESM2_ESM.docx]

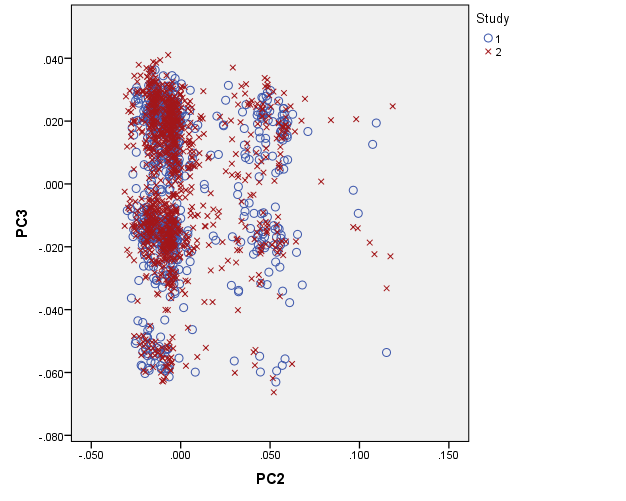


**PC1**

**PC2**


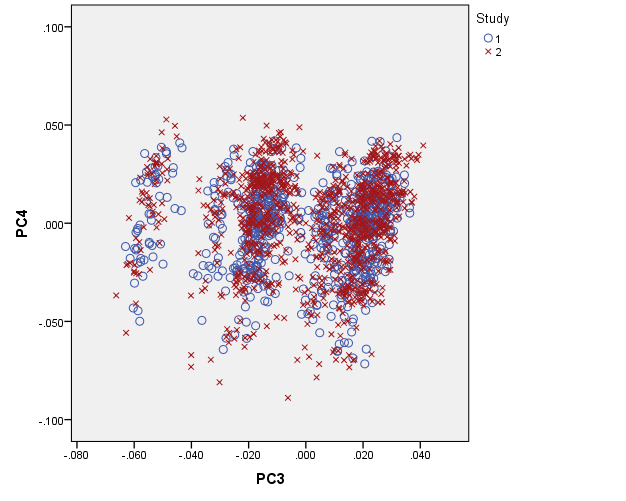


**PC2**

**PC3**

Supplementary Figure 1. Principle component analysis using 100 ancestry informative markers and comparing the cases and controls from the North Carolina Colorectal Cancer Study and the case and controls from the Chicago Colorectal Cancer Consortium. (A) PCA1 plotted against PCA2. (B) PC2 plotted against PC3.
